# Glenohumeral joint osteoarthritis is not associated with clavicle fractures in a large arthroplasty cohort

**DOI:** 10.1016/j.jor.2023.11.052

**Published:** 2023-11-22

**Authors:** Patrick J. Carroll, Mohamed Gaafer, David O'Briain, Darragh Hynes, Olivia Flannery, Hannan Mullett, Kieran O'Shea

**Affiliations:** a Department of Trauma & Orthopaedic Surgery, National Orthopaedic Hospital Cappagh, Cappagh Rd, Northside, Dublin 11, D11 EV29, Ireland; b Department of Trauma & Orthopaedic Surgery, Sports Surgery Clinic, Northwood Ave., Santry, Dublin 9, Ireland; c Department of Trauma & Orthopaedic Surgery, Blackrock Clinic, Rock Rd., Blackrock, Co. Dublin, A94 E4X7, Ireland; d Royal College of Surgeons in Ireland, 123 St. Stephen's Green, Dublin 2, D02 YN77, Ireland

**Keywords:** Shoulder, Clavicle, Fracture, Osteoarthritis, Arthroplasty, Replacement

## Abstract

**Introduction:**

A recent study based on a large osteological collection reported an association between clavicle fractures and osteoarthritis of the glenohumeral joint. No clinical study has yet addressed this potential association. Other radiographic parameters such as the critical shoulder angle have been associated with the risk of glenohumeral joint osteoarthritis. The primary outcome of this study was to determine if there is an association between glenohumeral joint arthritis and clavicle fractures. The secondary outcome was to determine the association between critical shoulder angle and glenohumeral joint arthritis in our patient cohort.

**Methods:**

We retrospectively analysed 572 consecutive shoulder arthroplasty surgeries. Osteoarthritis was the indication for 343 shoulder arthroplasties. 229 shoulder arthroplasties were performed due to another diagnosis such as trauma or fracture, cuff arthropathy, or revision surgery. Three fellowship trained consultant shoulder surgeons assessed the pre- and post-operative radiographs of all patients.

**Results:**

A clavicle fracture was suspected in 5/343 (1.5 %) shoulder arthroplasties performed due to osteoarthritis and 5/229 (2.1 %) shoulder arthroplasties performed for another diagnosis. Interobserver variability was assessed using a Fisher Exact test and showed no significant relationship between osteoarthritis and a fracture of the clavicle (p = 0.531). Critical shoulder angle results correlated with the previously published literature regarding critical shoulder angle and osteoarthritis and rotator cuff arthropathy.

**Conclusion:**

Clavicle fractures were not associated with glenohumeral osteoarthritis in our patient cohort of shoulder arthroplasty patients. Critical shoulder angle results were consistent with published literature. Further research in the form of prospective long term studies are needed to establish if any association exists between clavicle fractures and osteoarthritis of the glenohumeral joint.

**Level of evidence:**

Level III. Retrospective analysis.

## 1 Introduction

There is debate in the literature regarding the management of displaced clavicle fractures.^1^, ^2^, ^3^, ^4^ Historically clavicle fractures were almost always treated non-operatively.^5^ More recent literature has shown there may be a benefit to treating displaced clavicle fractures operatively.^6^ A recent systematic review and meta-analyses of 6 randomised control trials suggested there is not enough evidence to support routine operative treatment for all patients with a displaced midshaft clavicle fracture.^7^

It has been postulated that clavicle fractures are associated with arthritis of the glenohumeral joint.^8^ The study was carried out using 2899 cadaveric skeletons which enabled the authors to analyse almost 6000 clavicles and glenohumeral joints. This study's results could have a significant impact on the management of displaced clavicle fractures, with potentially more fractures being treated operatively. Fixation could occur more frequently to reduce the chances of developing symptomatic glenohumeral arthritis and reduce the chances of needing any joint replacement surgery of the glenohumeral joint.

We sought to add to the scant literature on this topic by adding a clinical study to see if there is a relationship between clavicle fractures and glenohumeral joint osteoarthritis. Shoulder replacement surgery is a definite endpoint for the treatment of symptomatic osteoarthritis.

## 2 Methods

Ethical approval was sought and granted to perform this research.

A retrospective analysis of 572 consecutive patients who underwent shoulder arthroplasty surgery was performed in the operating surgeon's institutions (Table 1). Databases were created including anonymised patient demographics and details. Pre- and post-operative radiographs were analysed by 3 fellowship trained shoulder and upper limb consultant surgeons.

**Table 1:** Patient characteristics.

	Total
Age, y (median)	73
Sex, Female (%)	409 (71)
Left Shoulder (%)	252 (44)
Diagnosis	
Primary Arthritis Group	343
Other Patient Group	229
CSA, (median)	37
Type of Shoulder Replacement	
aTSA	222
rTSA	289
Hemiarthroplasty	34
Revision	22
Other	5

Previous Clavicle Fracture	10

Age = Age at time of shoulder replacement surgery.

y = years.

CSA = critical shoulder angle.

Other patient group = cuff arthropathy, fracture, failed hemiarthroplasty.

aTSA = anatomic total shoulder arthroplasty.

rTSA = reverse total shoulder arthroplasty.

343 patients had a pre-operative diagnosis of osteoarthritis. 229 patients had a diagnosis such as trauma or fracture, rotator cuff arthropathy, or revision surgery. This group will be referred to as the ‘other patients’ group.

The primary outcome was whether a patient had suffered a clavicle fracture. These patients were subsequently stratified into patients with a previous clavicle fracture with primary osteoarthritis versus other causes for arthroplasty.

The critical shoulder angle (CSA) was obtained for each patient in the study. Student t-testing was performed to analyse CSA.

Inter-observer variability was calculated for the radiographic analysis performed by 3 fellowship trained shoulder and upper limb consultant surgeons. A Fisher Exact test was used to perform this.

A logistic regression was performed with osteoarthritis as the dependent variable and CSA, age and sex being independent variables.

All results were statistically analysed using the SPSS statistical package.^9^

## 3 Results

A clavicle fracture was suspected in 5/343 (1.5 %) shoulder arthroplasties performed due to osteoarthritis and 5/229 (2.1 %) shoulder arthroplasties performed for another diagnosis.

Interobserver variability was assessed using a Fisher Exact test (Table 2) and showed no significant relationship between osteoarthritis and a fracture of the clavicle (p = 0.531). Overall agreement was ‘fair agreement’ (Kappa = 0.352).^10^

**Table 2:** Interobserver variability was assessed using a Fisher Exact test.

	Fracture	Possible Fracture	No Fracture		Total
Consultant					
1	1	3	568		572
2	4	0	568		572
3	1	5	566		572

Fracture and Osteoarthritis Cross-tabulation					
			Osteoarthritis		
			No	Yes	Total
	Fracture	No	224	338	562
		Yes	5	5	10
	Total		229	343	572

Overall Agreement					
Kappa (95 % CI)					0.35 (0.35–0.53)
Fisher's Exact Test					
	Exact Sig. (2-sided)				0.531
	Exact Sig. (1-sided)				0.367

CI = Confidence Interval.

Sig. = significance.

**Fig. 1:**
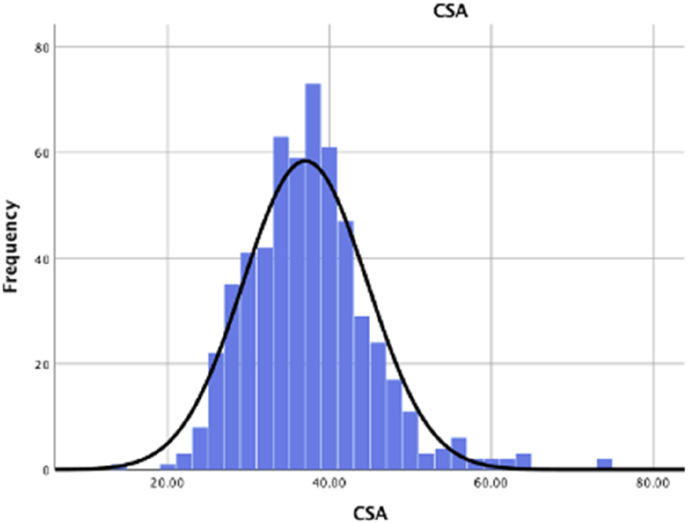
The critical shoulder angle was normally distributed in our patient cohort. CSA = critical shoulder angle.

**Fig. 2:**
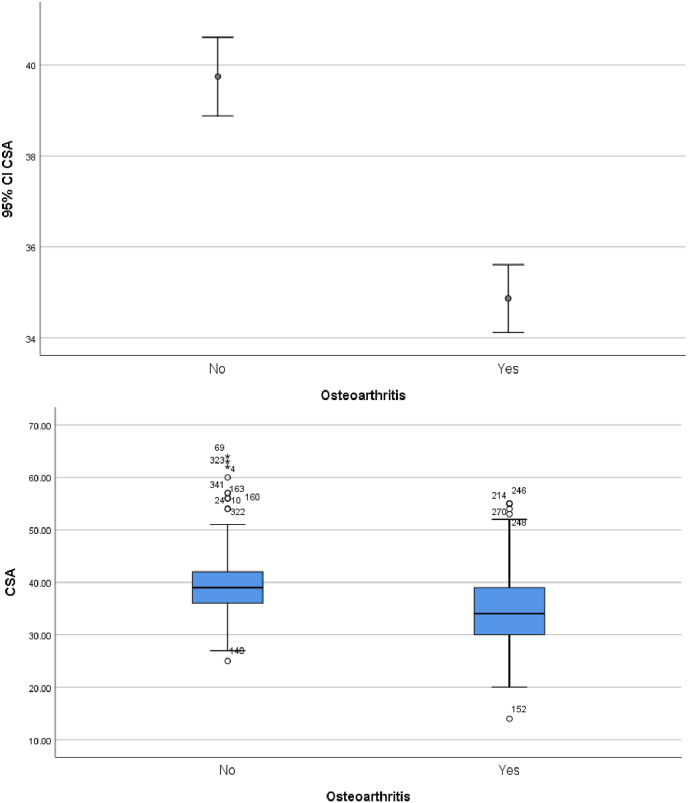
Student T-Test for assessing relationship between osteoarthritis and CSA, corresponding simple error bar and Boxplot.

**Fig. 3:**
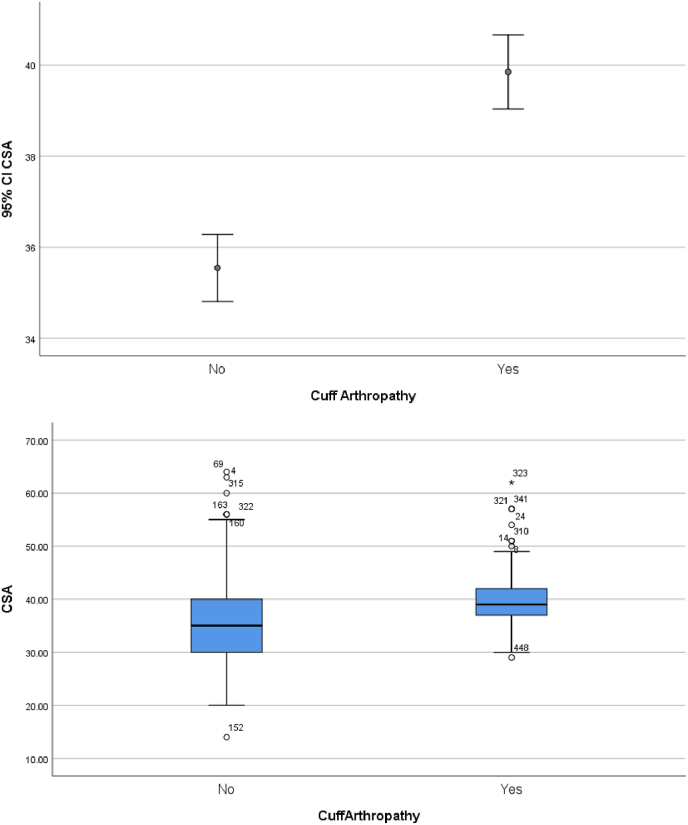
Student T-Test for assessing relationship between rotator cuff tear arthropathy and CSA, corresponding simple error bar and boxplot.

The critical shoulder angle was normally distributed in our patient cohort (Fig. 1). Student T-Tests and corresponding boxplots demonstrate critical shoulder angle results are lower for patients with osteoarthritis and higher for patients identified as having rotator cuff arthropathy (Fig. 2, Fig. 3).

**Fig. 4:**
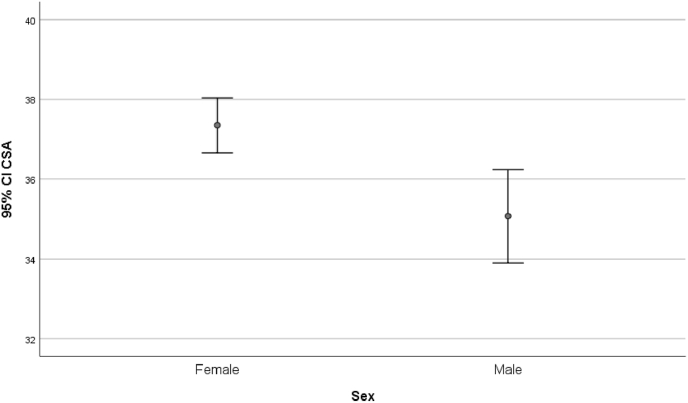
Association between CSA and sex demonstrated by T-Test and a simple error bar plot.

A logistic regression using osteoarthritis as the dependent variable and CSA, age, and sex as the independent variable was performed. This showed that for every unit increase in CSA there was approximately a 10 % reduction in the odds of osteoarthritis (Odds ratio (OR) = 0.899, p < 0.0001) (Table 3). Sex of the patient was not statistically significant. However, the CSA itself was then compared across both sexes to look for an association. This showed CSA is higher for females than males (p < 0.0001) (Fig. 4).

**Table 3:** Logistic regression model using osteoarthritis as dependent variable and CSA, age and sex as independent variables.

	OR	Sig.
CSA	0.899	p < 0.0001
Age	0.965	p < 0.0001
Sex	0.675	p < 0.08

Sig. = significance.

OR = odds ratio.

N = number of patients.

SD = standard deviation.

SEM = standard error of the mean.

CSA = critical shoulder angle.

CI = confidence interval.

## 4 Discussion

If a clavicle fracture was to be associated with glenohumeral joint arthritis of the shoulder, this would have significant consequences to how clavicle fractures are managed internationally in trauma and orthopaedic surgery facilities. The Cleveland cadaver study suggested that such association existed.^8^

This study is the first clinical study looking into this association since the publication of the Cleveland cadaver study. It is not possible for us to identify all clavicle fractures that have occurred in our patient population. However, an important end point for glenohumeral joint arthritis is a shoulder arthroplasty procedure. Only 1.5 % of patients in our study with glenohumeral joint osteoarthritis had suffered a suspected previous clavicle fracture. This shows that having a previous clavicle fracture and needing a shoulder arthroplasty procedure is extremely rare in our population.

The CSA for patients with osteoarthritis and rotator cuff arthropathy correlated with the international literature.^11^, ^12^, ^13^ This validates the CSA in our patient population for the first time to our knowledge. It also adds weight to the argument that a smaller critical shoulder angle is associated with osteoarthritis and a larger CSA is associated with rotator cuff arthropathy. Interestingly, our study showed that the older you get, the more likely you are to develop rotator cuff tear arthropathy. Also, of interest was the difference between critical shoulder angles for females and males. Females were more likely to have a higher CSA than males across all diagnoses in our patient cohort.

There are several limitations to our study. It is a retrospective study and by design this weakens the level of evidence of the study. The inter-observer variability was ‘fair’, and this highlights the difficulty in recognising previous clavicle fractures on radiographs even when performed by fellowship trained consultant surgeons. This is of interest in itself and further research regarding radiographic analysis of previous clavicle fractures should be performed to help identify previous fractures. Patient charts were not examined and therefore stratifying patient groups was made by x-ray alone. Differentiating between primary osteoarthritis or rheumatoid arthritis therefore was not possible. Subsequently, we have used primary osteoarthritis to encompass both osteo- and rheumatoid arthritis. Differentiating between osteoarthritis and rotator cuff arthropathy can be difficult radiographically and this should be acknowledged. Obviously, the best way to find out if clavicle fractures are correlated with osteoarthritis of the glenohumeral joint is to have shoulder radiographs for every person who has had a clavicle fracture in the past, however this is simply not practicable or feasible. Using a large arthroplasty database is a strong end point for patients with osteoarthritis of their glenohumeral joint and importantly symptomatic patients. We have not addressed patients who potentially have asymptomatic osteoarthritis of the glenohumeral joint. Measurement of the CSA was done based on antero-posterior shoulder radiographs. The authors would like to acknowledge that although these radiographs were performed by radiographers, that the AP shoulder radiographs cannot be guaranteed to be replicable between patients and therefore CSA may not be entirely accurate.

This is the first clinical study on this topic to be completed after the Cleveland paper which had found an association between clavicle fractures and osteoarthritis of the glenohumeral joint. This study includes a large shoulder arthroplasty patient cohort. This cohort has been validated by applying critical shoulder angles to the cohort with lower CSAs in the osteoarthritis group and higher CSAs in the rotator cuff arthropathy group. This is the first validation of the CSA in our patient population.

These findings could have other far-ranging implications from a medicolegal standpoint. Personal injury, disability and third-party claims occur frequently after a clavicle fracture. These findings would support the hypothesis that patients who have a clavicle fracture are no more likely than the general population without a clavicle fracture to have symptomatic glenohumeral joint arthritis requiring shoulder replacement surgery.

## 5 Conclusion

We have not seen any association between clavicle fractures and glenohumeral joint osteoarthritis. This is the first time the CSA has been validated in our patient population. For every unit increase in CSA there was approximately a 10 % reduction in the odds of osteoarthritis. We recommend a cautious approach to the management of clavicle fractures and we have not found any evidence to suggest clavicle fractures lead to symptomatic glenohumeral joint arthritis requiring a shoulder replacement.

## Ethical approval

Ethical approval granted by the Research Ethics Committee of The National Orthopaedic Hospital Cappagh. Reference number: NOHC/2020/ETH/SH-CEO/250.

## Financial remuneration/Disclaimer

None.

## Conflict of interest

We have no conflict of interest.
